# Editorial: Recent advancements in the dental biomaterials applied in various diagnostic, restorative, regenerative, and therapeutic procedures

**DOI:** 10.3389/fbioe.2022.1116208

**Published:** 2023-01-09

**Authors:** Mohammad Khursheed Alam, Kumar Chandan Srivastava, Mohd Fadhli Khamis, Adam Husein

**Affiliations:** ^1^ College of Dentistry, Jouf University, Sakaka, Saudi Arabia; ^2^ School of Dental Sciences, Universiti Sains Malaysia, Kota Bharu, Kelantan, Malaysia; ^3^ College of Dental Medicine, University of Sharjah, Sharjah, United Arab Emirates

**Keywords:** nanodentistry, silver nanoparticles, dental implant surface coating, osseointegration, guided bone regeneration, graphene, stem cell, modified MTA

## Recent advancements in the dental biomaterials applied in various diagnostic, restorative, regenerative, and therapeutic procedures

In recent times, dentistry has evolved in many directions at a very rapid pace. These advancements can be witnessed in every aspect of dentistry, including diagnosis, investigation, and therapeutic approaches, such as restorative, reparative, regenerative, and rehabilitative techniques. With improvements in investigative methodologies such as micro-computed tomography, scanning electron microscopy, spectroscopy, quantitative real-time polymerase chain reaction (PCR), cell line culture, immunohistochemistry, and laser scanning confocal microscopy (LSCM), the field’s understanding of diseases in terms of their etiopathogenesis and the available responses in the form of treatments have improved tremendously. Furthermore, nanotechnology has also contributed to a revolution taking place in various areas of the sciences, such as molecular biology, chemistry, and engineering. Dentistry is no exception to this: a vast amount of research is ongoing in this area, with existing dental biomaterials being modified to improve their physical, mechanical, and biological properties. Additionally, with the expanding need for treatment, new biomaterials are even being developed, while alterations to existing materials, such as restorative cements and obturating materials, are being improvised to overcome the shortcomings of their mechanical or chemical properties. There is demand for the development or improvisation of rehabilitating materials, such as dental implant surfaces and guided regeneration membranes, in order to make them more biocompatible, or to provide them with antibacterial properties or a cellular inductive nature.

In this Research Topic, a wide range of research papers have been published, discussing various aspects of the dental material sciences and taking the form of original research articles, narrative reviews, mini reviews, and case reports.

In a systematic review, Almehmadi compared the impact of titanium or zirconia implant surfaces coated with magnesium (Mg) to that of non-coated surfaces in terms of the success of implants. Based on the eligibility criteria and PICO statement, a total of 14 *in vitro* and animal studies were considered. Within the limitations of the review in terms of heterogenicity, the results of the *in vitro* studies showed an improvement in cell behavior, increased expression of various osteogenic markers such as alkaline phosphatase, and evidence of antibacterial properties in the case of the Mg-coated surfaces. The results of the animal studies also supported the use of Mg-coated surfaces, with these cases displaying characteristics such as high bone fill, enhanced bone–implant contact, and new bone formation.

In another systematic review and meta-analysis, Zhao et al. compared dental adhesives incorporating various plant extracts to adhesives without such additives in terms of their immediate strength of bonding with dentine. Furthermore, they also explored the difference in dentine strength between cases with and without the use of plant extracts as primers. Lastly, they also studied the influence of different types of plant extract and their use at varying concentrations. In consideration of the inclusion and exclusion criteria, a search strategy was devised that included all possible keywords. A total of 30 *in vitro* studies were considered for qualitative analysis, whereas 14 studies were analyzed quantitatively. It was concluded that there is a statistically significant improvement in immediate dentine-adhesive strength when adhesives with plant extracts are used. Additionally, improvement can be observed when plant extracts are used as primers. Among the plant extracts, 10% proanthocyanidine emerged as the most promising primer for improvement of the immediate bonding strength.

A original study by Xuan et al. aimed to compare a novel hierarchical intrafibrillarly mineralized collagen membrane (HIMCM) with the conventional therapeutic options for guided bone regeneration, such as collagen membrane and extrafibrillarly mineralized collagen. The newly fabricated membrane displayed superior physical properties (such as hydrophilicity and tensile strength) and chemical properties compared to the traditional options, and thus was found to mimic the microstructure of bone closely. Furthermore, HIMCM impregnated with bone marrow mesenchymal stem cells displayed enhanced signs of bone regeneration, such as proliferation and eventually differentiation into cells with a clear osteogenic fate. Additionally, in an animal experiment, the HIMCM was found to successfully fill a large surgical defect with bone analogous to neighboring bone in terms of density and architecture.

Another original piece of research by Abed et al. involved a comparative analysis of the chemical bond between glass ionomer cement (GIC) and the dentine of primary dentation, in which silver nanoparticles in three different concentrations (namely .2, .4, and .6%) were added to the GIC. The bond strength was enhanced by the addition of these nanoparticles, even at a low concentration of .4%.

Nanotechnology is a very promising avenue of researchin the dental biomaterial sciences, and many relevant research and review papers have been published in this Research Topic. Li et al. describe the details of research relating to graphene-based materials in a review paper. Graphene is a carbon-based nanomaterial and its derivatives possess excellent properties, such as biocompatibility, stimulation of cell differentiation, and anti-bacterial activity, which mean that it has various applications in dentistry. To mention a few, these include teeth whitening, adhesives, cements, coatings for dental implants, and tissue engineering, including bone and dental pulp. Yet another review, by Pushpalatha et al., discusses a versatile dental material, namely mineral trioxide aggregate (MTA). The paper describes the advantages of this material and its varied applications in dentistry. Additionally, the authors discuss the downsides of MTA, such as long setting time, discoloration, mud-like consistency, and poor handling characteristics; these have prompted new advancements, leading to the development of modified MTA. This subsequently developed materialsucceeds in overcoming the downsides of MTA and has better properties, such as improved setting time, enhanced compressive strength, microhardness, antibacterial activity, biocompatibility, regenerative ability, and suitability for local drug delivery.

Essentially, the aim of this Research Topic was to aggregate and disseminate work relating to current concepts and trends in dentistry, including but not limited to its diagnostic, restorative, and regenerative aspects. In addition to collecting and publicizing these developments, this Research Topic also attempts to bring multidisciplinary researchers together under a common roof. Furthermore, it also furnishes researchers and clinicians across the globe with an opportunity to share their innovative research and their perspectives on technological advancements in dentistry and how these can make a difference to patients’ quality of life. Lastly, this Research Topic will ignite the interest of a global readership in this Research Topic and stimulate further research ideas among them, as well as enhancing readers’ interest in this domain and assisting them in planning out their future research. This Research Topic covers the theme *via* all forms of research paper, including systematic reviews, meta-analyses, original research, narrative scoping reviews, and case series.

